# The bright side of boredom

**DOI:** 10.3389/fpsyg.2014.01245

**Published:** 2014-11-03

**Authors:** Andreas Elpidorou

**Affiliations:** Department of Philosophy, University of LouisvilleLouisville, KY, USA

**Keywords:** boredom, emotion, frustration, restlessness, weariness

Boredom *proneness* is commonly assessed and measured using self-report scales and questionnaires. The only full-scale measure of boredom that has been extensively used to assess boredom proneness is the Boredom Proneness Scale (BPS) (Farmer and Sundberg, 1986). BPS takes boredom proneness to be the tendency to experience boredom in a wide range of situations (Farmer and Sundberg, 1986). Although there is a close semantic relationship between the terms “tendency” and “disposition,” boredom proneness should not be understood as a dispositional state or property. A subject can possess the disposition to Φ even if the subject never actually Φ s. For that reason, I wish to suggest that one is prone to boredom not only if one possesses characteristics that make one susceptible to being bored, but also if one *frequently* experiences boredom^1^. Unlike a disposition that can remain hidden or non-actualized, boredom proneness, I hold, has visible and significant for the subject manifestations. Indeed, the frequent experience of boredom forms something akin to a pervasive lens through which the world is filtered. The boredom prone individual often and easily finds herself to be bored, even in situations that others, typically, find interesting and stimulating. Furthermore, she regularly becomes incapable of maintaining sustained attention, and interest in one's activities (Damrad-Frye and Laird, 1989; Eastwood et al., 2012; Malkovsky et al., 2012), she lacks excitement for, or can find no purpose in, what she is doing (Barbalet, 1999; Fahlman et al., 2009; van Tilburg and Igou, 2012), and she easily becomes frustrated, restless, or weary by either stimuli-poor or challenging situations (Farmer and Sundberg, 1986). Boredom proneness is associated with a plethora of significant bodily, psychological, and social harms (Vodanovich, 2003). Boredom proneness is positively correlated with depression and anxiety (Ahmed, 1990; Blaszczynski et al., 1990; Sommers and Vodanovich, 2000; Goldberg et al., 2011; LePera, 2011), anger and aggression (Gordon et al., 1997; Rupp and Vodanovich, 1997; Dahlen et al., 2004), a lower tendency to engage in and enjoy thinking (Watt and Blanchard, 1994; Seib and Vodanovich, 1998), a propensity to make mistakes in completing common tasks (Wallace et al., 2002), poor interpersonal and social relationships (Leong and Schneller, 1993; Watt and Vodanovich, 1999), lower job and life satisfaction (Farmer and Sundberg, 1986; Kass et al., 2001), problem gambling (Blaszczynski et al., 1990; Mercer and Eastwood, 2010), and drug and alcohol abuse (Lee et al., 2007; LePera, 2011).

Boredom proneness ought to be distinguished from the state of boredom, i.e., the actual experience of boredom (cf. Fenichel, 1953; O'Hanlon, 1981; Neu, 1998; Fahlman et al., 2013). Even though boredom proneness is predicated on the antecedent experience of boredom—viz., one cannot be said to be prone to boredom if one never experienced boredom—the very experience of boredom does not entail boredom proneness. One can be in a state of boredom without one necessarily being prone to boredom. Understood as a psychological state that is not necessarily the manifestation of boredom proneness, boredom is an aversive, transient state that can be often easily alleviated. It can also be a rather common and mundane experience. For instance, most of us experience boredom while waiting in line to pay for our shopping, when our bus or train is delayed, or when we have to endure the same conversation, lecture, or TV show over and over again. Often, boredom is situational: it is brought about by the unchallenging, monotonous, or repetitive situations in which we find ourselves (O'Hanlon, 1981). As a result, boredom can be allayed by a mere change in setting. Boredom, however, need not always be situational. Depending on our moods, desires, and attitudes, situations that are ostensively meaningful and challenging could also bring about the state of boredom (Geiwitz, 1966; Mikulas and Vodanovich, 1993); conversely, situations that are repetitive, monotonous, and utterly humdrum do not have to lead to the experience of boredom (Perkins and Hill, 1985; DeChenne and Moody, 1988).

Theoretical discussions of the state of boredom have been predominantly one-sided. Notwithstanding a few notable exceptions (Nietzsche, 1974, p. 108; Brodsky, 1995; and Russell, 1996, pp. 48, 52), the literary and philosophical history of boredom consists of a series of attempts to articulate boredom's distinctively negative nature. Boredom is thought to be disruptive and harmful (Dostoevsky, 1962, p. 418; Stendhal, 1975, p. 211; Burton, 1621/2001, p. 390; Pessoa, 2002, p. 76; Novalis in Donehower, 2012, p. 48), a great source of unhappiness and suffering (Schopenhauer, 1966, pp. 204, 312–313, 321; Beckett, 1978; Nisbet, 1982, p. 26; Kierkegaard, 1987, pp. 37, 285–286; Fromm, 1990; Schachtel, 2001, p. 183) and, ultimately, a state that hinders the development of one's intellectual, social, and even moral capacities (Dante's *Purgatorio*, XVII-XIX; Cassian, 2000; Sinkewicz, 2003; Pascal, 2004, pp. 40, 163). Boredom, as “the root of all evil,” is a problematic state (Kierkegaard, 1987, p. 286); it has very few, if any, redeeming characteristics^2^.

Despite its impressive historical backing, the view that boredom is entirely negative should be rejected. Recent empirical work on boredom, taken in tandem with theoretical considerations about its nature and character, suggest a rather different picture of the state of boredom (Barbalet, 1999; van Tilburg and Igou, 2012; Bench and Lench, 2013; Gasper and Middlewood, 2014). In broad strokes, the picture is as follows: on account of its affective, volitional, and cognitive aspects, boredom motivates the pursuit of a new goal when the current goal ceases to be satisfactory, attractive, or meaningful to the agent. Boredom helps to restore the perception that one's activities are meaningful or significant. It acts as a regulatory state that keeps one in line with one's projects. In the absence of boredom, one would remain trapped in unfulfilling situations, and miss out on many emotionally, cognitively, and socially rewarding experiences. Boredom is both a warning that we are not doing what we want to be doing and a “push” that motivates us to switch goals and projects. Neither apathy, nor dislike, nor frustration can fulfill boredom's function (Goldberg et al., 2011). In what follows, I elaborate on this picture of boredom and make a case for its value and significance.

But first, more needs to be said about the very nature of the state of boredom. In terms of its affective (or qualitative) character, boredom is an aversive state that is characterized by feelings of dissatisfaction, restlessness, and weariness. Although additional feelings might be present during the state of boredom (e.g., mild frustration), the aforesaid feelings are essential to boredom. Not only subjects who experience boredom report on having these feelings, but also, and most importantly, the lack of any one of these feelings would result in a psychological state that is different than boredom (van Tilburg and Igou, 2012; Fahlman et al., 2013; cf. Thackray et al., 1975; Hill and Perkins, 1985; Perkins and Hill, 1985; O'Brien, 2014). Boredom without dissatisfaction cannot be boredom: to be satisfied with one's situation is to cease to be bored with the situation. Restlessness and weariness are equally essential. When one is bored one is not content with one's situation. The state of boredom is one from which we seek to escape. While bored, one is thus restless, for one is not content with one's current situation and one wishes to be doing something else (van Tilburg and Igou, 2012). Lastly, the experience of boredom involves weariness. To experience boredom is to experience a certain kind of weariness or mental fatigue. The boring, in other words, tires us.

The experiential character of boredom includes more than its affective aspects. That is to say, boredom is not merely a sensual or a qualitatively state. While bored one has certain thoughts about one's situation or object of attention. Often, for example, one thinks that the situation in which one finds oneself is bereft of significance or meaning (van Tilburg and Igou, 2012), or that what one is currently doing is incongruous with one's plans. Furthermore, while bored one has a desire, often quite strong, to engage in a different and more satisfying activity. One also has a difficulty maintaining and focusing one's attention (Bernstein, 1975; Fisher, 1993; Martin et al., 2006; Eastwood et al., 2012). One even experiences a slow passage of time (Fenichel, 1953; O'Connor, 1967; Hartocollis, 1972; Conrad, 1997; Martin et al., 2006).

In a state of boredom, the world is revealed to oneself in a particular and indeed striking fashion (Elpidorou, forthcoming). The world appears not only to be uninteresting, but also distant, foreign, and often unyielding. Boredom contributes to a loss of value, significance, or meaning. The world of boredom is, in a sense, not *our* world: it is not the world that is in line with our projects and desires. Our current situation does not attract us; we do not feel compelled to engage with it. The weariness that we experience while bored, compounded with the perception of a slower passage of time, makes the character of boredom all the more aversive (Sackett et al., 2010). Being in a state of boredom feels like being emotionally trapped: the unsatisfying situation with which we are engaging seems to last longer; it appears to be removed from our concerns; and we feel weary. Crucially, however, boredom is not a state of equilibrium. We are not content in it. We feel restless and wish to be doing something else. We desire escape from boredom. In turn, while bored it is difficult for us to focus our attention on features of the present situation. Our mind wanders and alternative goals and situations suddenly become salient to us. If boredom is likened to an emotional trap, it is a trap that due to its own character fortunately “pushes” us to escape from it.

The significance and value of boredom should now be apparent. First, boredom is informative: it tells us something both about the world and about ourselves. While bored, our situation is disclosed to us as unfulfilling, uninteresting, unchallenging, or non-stimulating. On account of this disclosure, boredom also informs us of our own goals, interests, and even self-perceived wellbeing. Being bored means that we are currently engaged not only in an uninteresting or unchallenging situation, but also in a situation that fails to meet our expectations and desires. Boredom is a state that is about ourselves as much as it is about the world.

Second, and most importantly, the negative and aversive experience of boredom acts as a force that motivates us to pursue a goal that appears to us to be more stimulating, interesting, challenging, or fulfilling than the goal that we currently pursue (Barbalet, 1999; van Tilburg and Igou, 2012; Bench and Lench, 2013). In an episode of boredom, our current goal or situation is perceived as unpleasant and unappealing, often as devoid of meaning or significance; on the contrary, alternative goals and situations are made salient and appear to us to be attractive. Boredom thus facilitates the pursuit of alternative goals: it “pushes” us *out* of this non-stimulating, uninteresting, or unchallenging situation and *into* another. In motivating us to pursue a situation that is different from our current one, boredom ultimately promotes the restoration of the perception that one's activities are meaningful and congruent with one's overall projects (Locke and Latham, 1990; Sansone et al., 1992; Heine et al., 2006). What alleviates boredom is not simply a change of activity. Rather, what alleviates boredom is a change from an uninteresting, unfulfilling, or non-stimulating situation to one that is perceived by the agent to be satisfactory and in line with her plans and wishes.

Given its twofold function, boredom is best understood as a state that monitors and regulates our behavior. It informs us when we are out of tune with our interests, and, on account of its aversive character, motivates us to engage in situations that are perceived by us as fulfilling or meaningful. Boredom is important. It promotes our interests by trying to keep us in touch with what we care about. It safeguards us from emotional traps and long-term dullness.

## Conflict of interest statement

The author declares that the research was conducted in the absence of any commercial or financial relationships that could be construed as a potential conflict of interest.

## Footnotes

^1^In Fahlman et al. (2013), a disjunctive account of boredom proneness is offered. “When a person declares herself to be prone to boredom, she is claiming (i) that she *frequently* experiences boredom, or (ii) that she *possesses the qualities that predispose* her to experience boredom *given the right circumstances*” (81; Roman numerals added). My description of boredom proneness is conjunctive: both (i) and (ii) are taken to be necessary in order for an individual to be prone to boredom. If (ii) was a sufficient condition for boredom proneness, then one could be said to be prone to boredom even if one never experienced boredom (e.g., one possesses the relevant characteristics but one never finds herself in circumstances that permit those characteristics to bring about the experience of boredom). If (i) was sufficient, then accidental environmental (or exogenous) conditions could render an individual prone to boredom, even if the individual possesses none of the characteristics that predispose her to experience boredom.

^2^More recent theoretical discussions of boredom do not diverge greatly from the practice of treating boredom as a problem or malaise. See e.g., Healy (1984, pp. 11, 75) and Svendsen (2004, pp. 16, 154) Even Brodsky, who sets out to defend boredom, describes boredom as an “incurable malaise” (1997, p. 104).
